# Rotation of anatomical breast tissue expanders: a comparison of microtextured surfaces from different manufacturers

**DOI:** 10.1016/j.jpra.2026.07.040

**Published:** 2026-08-08

**Authors:** N Heine, A Eigenberger, L Prantl, A Wiesmeier

**Affiliations:** Department of Plastic, Reconstructive and Hand Surgery, University Hospital Regensburg, Franz-Josef-Strauss-Allee 11, 93053 Regensburg, Germany

**Keywords:** Breast reconstruction, Tissue expander, Implant rotation, Microtextured surface, Anatomical implant, Breast cancer surgery

## Abstract

**Background:**

Anatomical tissue expanders are widely used in two-stage breast reconstruction following mastectomy. In recent years, microtextured implant surfaces have largely replaced macrotextured devices in order to reduce the risk of capsular contracture while maintaining tissue integration. However, rotation of anatomical expanders remains a clinically relevant complication that may compromise reconstructive outcomes. Comparative data regarding the rotational stability of different microtextured surfaces are limited.

**Methods:**

This retrospective cohort study included patients who underwent mastectomy followed by two-stage breast reconstruction using anatomical tissue expanders between January 2017 and December 2024 at two hospitals. Microtextured expanders from Mentor (Siltex surface) and Polytech (POLYtxt surface) were compared. The primary endpoint was expander rotation ≥45°, assessed intraoperatively at the time of expander exchange. Secondary endpoints included reconstruction outcomes, complications, and potential risk factors such as radiotherapy, chemotherapy, smoking status, and body mass index (BMI).

**Results:**

A total of 164 patients with 181 reconstructed breasts were included (74 Siltex, 107 POLYtxt). Rotation ≥45° occurred significantly more frequently in the Siltex group (33.8%) than in the POLYtxt group (11.2%) (χ²(1)=13.7, p=0.0002). The relative risk of rotation with Siltex expanders was 3.01 (95% CI 1.62–5.61). Patient demographics and expander volumes were comparable between groups. Despite a higher rate of adjuvant radiotherapy in the POLYtxt group, rotation remained less frequent than in the Siltex group. Smoking and chemotherapy were associated with higher rotation rates in the Siltex group, whereas no comparable increase was observed in the POLYtxt group. Other complication rates were similar between groups.

**Conclusions:**

Siltex microtextured expanders demonstrated a significantly higher rate of clinically relevant rotation compared with POLYtxt expanders. These findings suggest that microarchitectural differences between surfaces classified as microtextured may influence rotational stability. When Siltex expanders are used, additional stabilization measures such as fixation tabs should be considered.

## Introduction

A few years after Cronin et al.'s first publication on silicone-based breast implants in 1964, Radovan developed a silicone expander for tissue expansion after mastectomy to prepare for breast reconstruction.^1^ While the first generation of silicone implants had a smooth shell, textured surfaces were first used in the 1970s^2^ to reduce capsular fibrosis and improve integration with surrounding tissue. This surface design enabled the creation of an anatomical teardrop shape to reconstruct a natural breast following a complete mastectomy. In particular, macrotextured implants promised high rotational stability and low rates of capsular fibrosis.^3^

As early as 1997, Keech et al. described a case of anaplastic large cell lymphoma (ALCL) associated with breast implants.^4^ In 2011, the U.S. Food and Drug Administration (FDA) published a report on breast implant-associated anaplastic large cell lymphoma (BIA-ALCL) in patients with breast implants.^5^ Since 2012, cases of BIA-ALCL have been recorded in the FDA's "PROFILE" registry in collaboration with U.S. professional associations.^6^ A higher number of cases have been diagnosed in patients with macrotextured implants with a roughness greater than 50 µm according to ISO 14607:2018.^7^ Therefore, there is a trend toward microtextured or smooth implants.

After the 2019 withdrawal of macrotextured Biocell implants,^8^ many institutions have abandoned macrotextured implants in favor of smooth or nano/microtextured products.^9^

In light of this development, the rotational stability of the various surface textures has become particularly relevant. For implants in situ, the extent of rotation relative to the original position can only be imprecisely estimated. The most precise measurement of rotation relative to the correct position is possible in the case of a two-stage reconstruction with a temporary expander because the misplacement can be determined precisely when the expander is removed, which is always necessary when exchanging it for the definitive implant.

In our study, we compared the rotational behavior of microtextured temporary expanders from Mentor (Mentor Worldwide LLC, Santa Barbara, CA, USA) with "Siltex" texturing and microtextured expanders from Polytech (Polytech Health and Aesthetics, Dieburg, Germany) with "TXT" surfaces.

The study aimed to investigate the rotation behavior of temporary breast expanders from two different manufacturers whose surfaces are classified as "microtextured" with comparable pore sizes and evaluate other patient- and product-related parameters.

## Materials and methods

This study was conducted with the approval of the University of Regensburg's ethics committee (25-4330-104). A retrospective record study was performed at Caritas Hospital St. Josef in Regensburg, in the Department of Plastic, Hand, and Reconstructive Surgery, as well as in the Gynecology Department of the Clinic of Neumarkt/Oberpfalz.

Between January 1, 2017, and December 31, 2024, a total of 164 patients underwent mastectomy and reconstruction with tissue expanders at these two clinics. The expander was replaced with a silicone implant or a free flap afterwards. The clinic in Neumarkt used tissue expanders manufactured by Mentor LLC (Santa Barbara, CA, USA) with Siltex texturing, while Caritas Hospital St. Josef in Regensburg used temporary expanders manufactured by Polytech Health & Aesthetics (Dieburg, Germany) with POLYtxt texturing (surface roughness of 35 µm according to ISO 14607:2018). Both manufacturers define the aforementioned surfaces (Siltex and POLYtxt) as "microtextured" according to the ISO 14607 classification. Mentor specifies a pore size of 29.5 to 36.1 µm and an "imprint" embossing technique for Siltex surfaces, whereas the POLYtxt surface is produced by vulcanizing the silicone shell with the thermal decomposition of ammonium carbonate salts.^10^ This results in a microtextured surface with an average roughness of approximately 35 µm.^11^

In all cases, a submuscular implant pocket was prepared according to the planned expander size. The expanders were partially filled after deflation, then inserted without rotation after the gloves and instruments were changed. The expanders were not stabilized with fixation sutures. A Redon drain was inserted into the implant pocket. The muscle pocket was closed with absorbable suture material. All expander insertions and removals were performed by board-certified specialist surgeons at each respective centre. While the operating surgeon at explantation was not necessarily the same individual who performed the initial insertion, all surgeons involved held specialist-level qualification. The surgeon who performed the explantation also assessed the degree of rotation intraoperatively; this assessment was additionally confirmed by the assisting resident surgeon present during the procedure.

Postoperatively, patients wore a compression bra for at least six weeks. Filling began in the first or second week and rapidly advanced to the nominal volume by injection every one to two weeks.

Data was retrospectively collected in both clinics, including an evaluation of the patient's demographic data, previous illnesses, relevant previous operations, and surgical data, especially expander rotation and subsequent complications, if any. Additionally, risk factors such as nicotine abuse and previous radiotherapy were recorded. All data were listed in tabular form and then statistically analyzed.

The inclusion criteria were patients aged 18 years or older who underwent mastectomy with two-stage reconstruction using Mentor Siltex or Polytech POLYtxt expanders between January 1, 2017, and December 31, 2024, and who had available surgical and follow-up records. Exclusion criteria included the use of expanders from other manufacturers or surface textures; primary autologous reconstruction without an expander; revision cases with insufficient baseline data; and missing consent for data use.

The primary endpoint of this study was expander rotation, which was defined as a documented rotation of at least 45°. The metric of interest was the proportion of patients with rotation in each group (Mentor Siltex versus Polytech POLYtxt). Effect estimates were expressed as relative risks (RR) and absolute risk differences, each with 95% confidence intervals (CI).

The secondary endpoints included the type of definitive reconstruction (implant-based, autologous, or none), other complications (such as capsular contracture, seroma, infection, or expander damage), and the time to definitive reconstruction.

Exploratory analyses evaluated the influence of factors such as adjuvant or neoadjuvant radiotherapy, body mass index (BMI), smoking status, expander shape and volume, and the occurrence of complications.

The primary hypothesis tested was that there is no difference in rotation rates between Siltex (Mentor) and POLYtxt (Polytech) expanders (H0). The alternative hypothesis (H1) was that the rotation rates differ between the groups.

Statistical analyses were performed using R version 4.5.2 (R Foundation for Statistical Computing, Vienna, Austria). Microsoft Excel 365 (Microsoft Corporation, Redmond, WA, USA) was used for data management and preliminary descriptive analyses. Continuous variables are presented as mean ± standard deviation.

Categorical variables were reported as absolute numbers and percentages and compared using the Pearson chi-square test when all expected cell counts exceeded five. When expected cell counts were less than five, Fisher’s exact test was applied.

The primary endpoint was expander rotation ≥45°. Relative risks (RR) with corresponding 95% confidence intervals (CI) were calculated to quantify the association between expander surface type and rotation. Confidence intervals for the relative risk were computed using the logarithmic transformation method. All tests were two-sided, and a p-value < 0.05 was considered statistically significant.

## Results

### Baseline and demographic data

A Total of 164 patients and 181 breasts were included in summary; 65 patients with 74 breasts at the Clinic of Neumarkt, who were reconstructed with Mentor expanders/Siltex surface, 99 patients and 107 breasts at Caritas Hospital St. Josef in Regensburg, who were reconstructed with Polytech expanders/TXT surface.

9 patients at Clinic of Neumarkt and 8 patients at Caritas Hospital St. Josef underwent surgery on both sides.

In the Siltex group, the average age at the time of surgery was 54.1 ± 10.3 years, the average BMI was 25.2 ± 4.8 kg/m^2^. 18 patients had a history of smoking. In the TXT group, the average age at time of surgery was 50.7 ± 10.1, the average BMI was 25.1 ± 4.3 kg/m^2^. 14 patients were smokers.Tables 1, 6.

**Table 1:** In- and exclusion criteria.

Inclusion criteria	Exclusion criteria
Two-staged reconstruction using Mentor Siltex or Polytech POLYtxt expander	Patients in need of care / particularly vulnerable patients
Patients > 18 years of age	Reconstruction with expander from other manufacturers or surface
Consent for data use	Primary implant-based breast reconstruction
Complete and sufficient data	Primary reconstruction with autologous tissue

The results are also shown in Table 2. Table 3 shows information about tumor therapy additional to performed mastectomy. In Table 4 lists surgery-related complications.

**Table 2:** Patient-related baseline data.

	Mentor Siltex	Polytech POLYtxt
Average age at time of surgery	54.1 ± 10.3	50.7 ± 10.1
Average BMI in kg/m^2^	25.2 ± 4.8	25.1 ± 4.3
Average height in cm	166.6 ± 5.9	166.8 ± 6.5
Average weight in kg	69.8 ± 13.1	69.9 ± 13.5
Smoking	18 (24.3%)	14 (13.1%)

**Table 3:** Tumor- therapy data.

	Mentor Siltex (n=74)	Polytech POLYtxt (n=107)
Chemotherapy	45 (60.8%)	61 (57.0%)
Radiation	11 (14.9%)	56 (52.3%)
Antihormone therapy	50 (67.6%)	79 (73.8%)

**Table 4:** Surgery- related complications.

	Mentor Siltex (n=74)	Polytech POLYtxt (n=107)
Increased seroma	3 (4.1%)	3 (2.8%)
Hematoma	0	2 (1.9%)
Infection/Redness	1 (1.4%)	10 (9.35%)
Resistant pain	0	2 (1.9%)
Capsular fibrosis	1 (1.4%)	12 (11.2%)
Defect of expander	2 (2.7%)	7 (6.5%)

The rate of implant defects did not differ significantly between the Siltex group (2.7%) and the POLYtxt group (6.5%) (Fisher’s exact test, p = 0.31)

As shown in Table 5, the average resection weight in the Siltex group was 486.6 ± 281.9 g and 578.3 ± 411.2 g in the TXT group. The average expander volume was 460.5 ± 124.9 cc in the Siltex and 460.6 ± 113.6 cc in the TXT group.

**Table 5:** Rotation of expander.

	Mentor Siltex (n=74)	Polytech POLYtxt (n=107)
Rotation ≥45°	25 (33.8 %)	12 (11.2 %)

**Table 6:** Surgery related baseline data.

	Mentor Siltex (n=74)	Polytech POLYtxt (n=107)
Resection weight (g)	486.6 ± 281.9	578.3 ± 411.2
Expander volume (cc)	460.5 ± 124.9	460.6 ± 113.6
Duration until explantation (d)	268.6 ± 119.6	305.6 ± 165.4
Final reconstruction with silicone implant	70 (94.6%)	65 (60.8%)
Final reconstruction with free flap	0	27 (25.2%)
No final reconstruction	4 (5.4%)	15 (14.0%)

Siltex surfaced expanders were in situ for an average of 268.6 ± 119.6 days, while polytextured expanders were explanted after 305.6 ± 165.4 days in average.

**Fig. 1:**
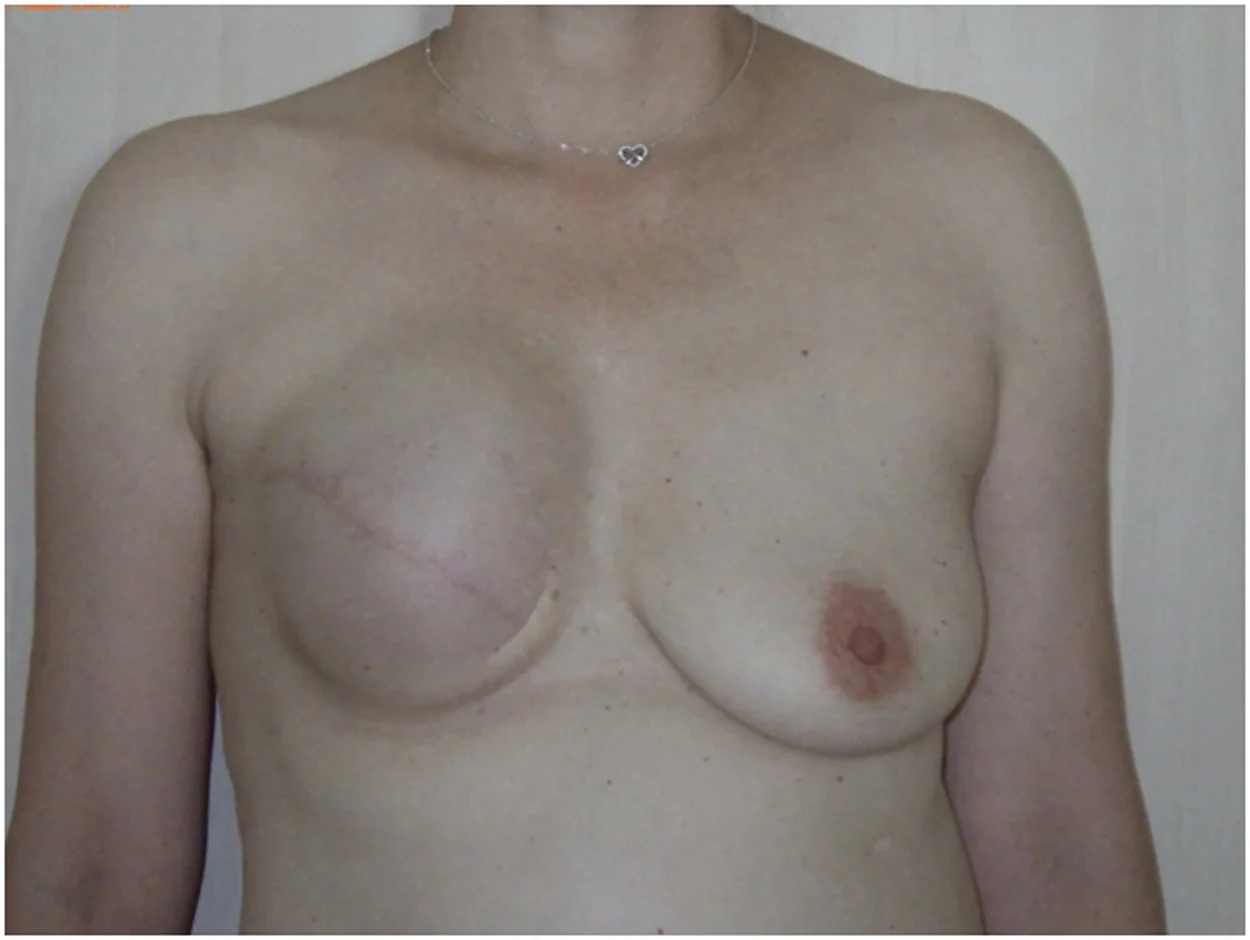
Clinical picture of a rotated Mentor Siltex expander, already visible in the image.

**Fig. 2:**
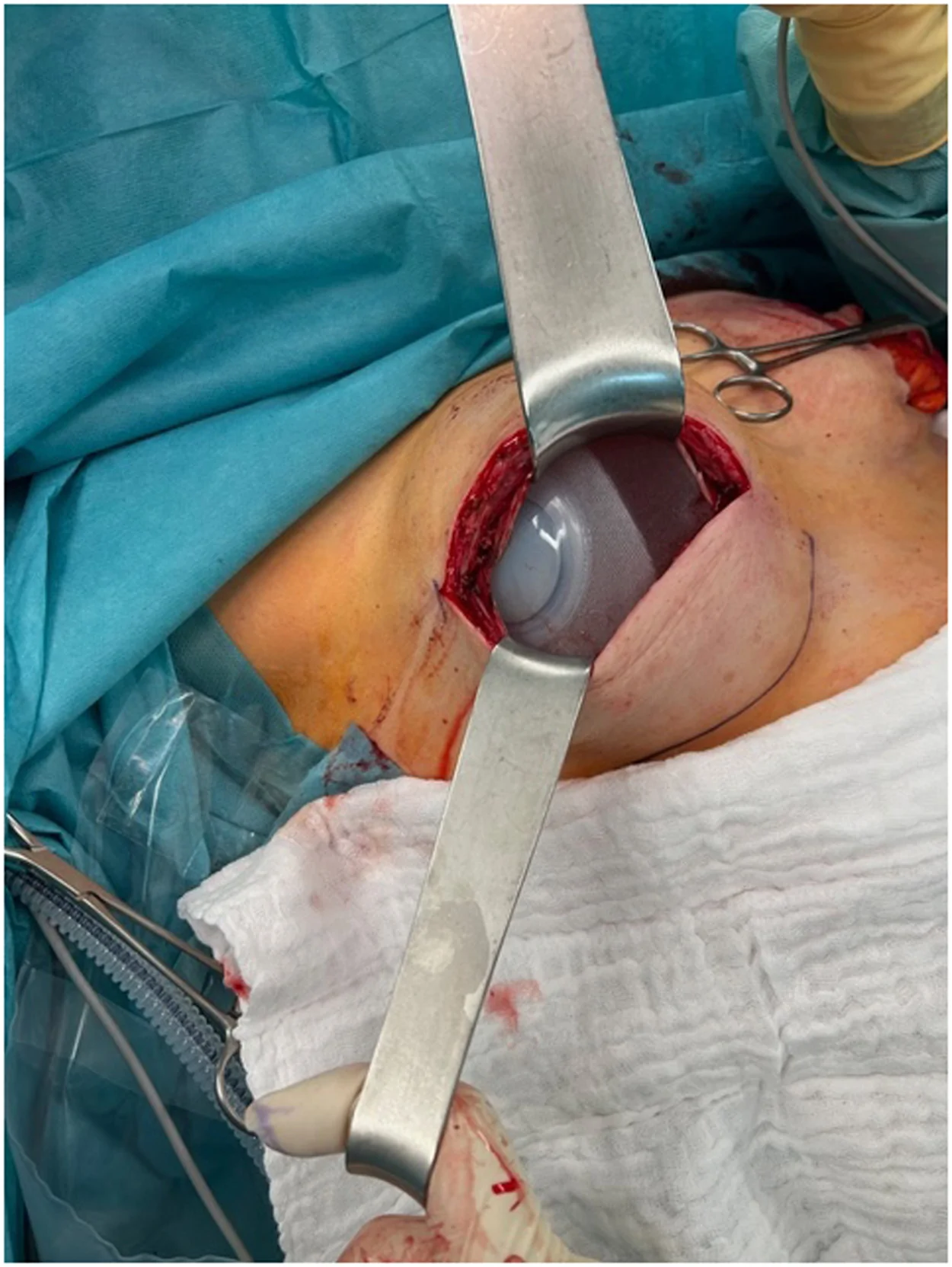
Intraoperative confirmation of expander rotation in the patient`s situs. The valve is rotated 90° laterally (Left in the picture = lateral to the patient).

Rotation was evaluated at time of explantation. Overall, 37 expanders exhibited clinically relevant rotation ≥45°, comprising 25 Siltex and 12 POLYtxt devices (Table 5). The incidence of rotation was significantly higher in the Siltex group (33.8%) than in the POLYtxt group (11.2%) (χ²(1) = 13.7, p = 0.0002). The relative risk of rotation with Siltex expanders was 3.01 (95% CI 1.62–5.61). Figs. 1 and 2 show a patient example with a rotated expander.

After explantation of the expander, 70 breasts of the siltex group and 65 breasts of the TXT group were reconstructed by silicone implants. 15 breasts in the TXT and 4 breasts in the Siltex group got no reconstruction, the expander was removed. Reasons were infections in 7 cases (n=6 TXT Group, n=1 Siltex Group), resistant pain and capsular fibrosis in 4 cases (TXT group).

In the Siltex group no free flap reconstruction was performed. In the TXT Group, 27 breasts were reconstructed via free flap (n=26 DIEAP flap, n=1 TMG flap).

## Discussion

Reconstruction after mastectomy as a result of malignant diseases of the female breast isone of the most common procedures in plastic surgery. Since the 1980s, techniques using autologous tissue and implant-based reconstructions have been developed, with an increase of implant-based procedures in recent years, particularly in the USA.^12^ In addition to direct-to-implant reconstructions and the less frequent use of permanent expander prostheses, two-stage reconstruction of the breast with a temporary expander and subsequent replacement with a permanent implant is one of the most common techniques for breast reconstruction.

Since anatomical expanders and implants are usually performed following mastectomy, rotation often leads to deformities that require revision. Especially since the increased incidence of BIA-ALCL in connection with macrotextured implants,^13^ there has been a trend toward micro- and nanotextured surfaces. Therefore, the rotational stability of anatomical breast implants has to be regarded as an important quality feature.

Rotation of implants appears to occur more frequently than described in the literature. One reason could be that malposition is usually assessed clinically without being able to determine the exact position of the implant. In a single-center study with a follow-up period of 18.8 months involving 1,527 patients, Montemurro et al.^14^ found clinical rotation in 1.8% of cases. Gary et al.^15^ assessed rotation, similar to our study, during surgical revision and found rotation of 30° or more in 45% of 51 patients. It should be noted, however, that their study only included women with indication for revision. Schoberleitner et al.^16^ compared the reaction to different surface textures in breast expanders (Siltex vs. SmoothSilk). They found a stronger tissue reaction with Siltex, although the comparison product had a nano-textured surface and therefore a pore size that was not directly comparable. Weltz et al.^17^ examined the rotation behavior of 653 patients after aesthetic augmentation with anatomical, microtextured implants and found clinically relevant rotation of the implants in 2.6% after primary augmentation and in 3.2% after secondary augmentation.

In our study, we were able to record 107 expanders with a TXT surface in 99 patients and compare them with 74 expanders (65 women) with a Siltex surface. The degree of rotation was measured intraoperatively after marking at the most inferior point of the implant still in situ; a rotation of at least 45° was considered relevant. This study is one of the few investigations of implant rotation in situ, while most publications assess the extent of rotation based solely on clinical appearance.

The most striking result was, that clinically relevant rotation ≥45° occurred in 25 of 74 expanders (33.8%) in the Siltex group and in 12 of 107 expanders (11.2%) in the TXT group. This difference was statistically significant (p < 0.001) and corresponded to a threefold increased relative risk with Siltex devices (RR 3.01, 95% CI 1.62–5.61).

Since the pore size of the two surfaces examined in this study were comparable, further parameters had to be examined that could be seen in connection with the different rotation rates. One important procedural variable is the position of the implant pocket and whether the muscle was fully closed following expander insertion. In our cohort, all expanders were placed subpectorally, and the muscle pocket was closed using a continuous absorbable suture. As pocket position and closure technique were therefore identical across both groups, this factor can be excluded as a potential confounder of the observed differences in rotation rates between the Siltex and POLYtxt groups.

The biometric data of the patients were comparable, with a height of 166.6 cm (Siltex), 166.8 cm (TXT) and a weight of 69.9 kg (TXT) and 69.8 kg (Siltex; each BMI of 25) respectively; the age of the TXT group was slightly younger at 51 years than in the Siltex group at 54 years. Similarly, the target volume of the expanders used was almost identical at 409 ml (TXT) and 460 ml (Siltex), respectively, while the weight of the tissue removed during mastectomy was lower in women who underwent reconstruction with a Siltex expander (486.6 g) than in patients who underwent reconstruction with a TXT expander after resection of 578.3 g of glandular tissue.

Kern et al^18^ found that patients with a higher BMI were more prone to implant rotation. However, since both the body mass index and the average expander size were comparable in both groups, it can be assumed that the higher breast weight in mastectomy did not have a significant influence on the reconstructive result.

The in-situ time of the expanders used was slightly shorter in the Siltex group at 268 days than in the TXT group at 305 days. Fujii et al^19^ found an increased risk of rupture with long-term indwelling of tissue expanders beginning at 1.5 years after insertion and 32.6% by the third year; they recommended switching to the definitive implant after one year. There is no evidence in the literature to suggest that a shorter indwelling period for the expanders would have a negative effect on rotation behavior. It is unclear to what extent the risk of rotation increases with longer in-situ time of the expander; however, this is contradicted by the fact that in our study the TXT group had a lower rotation rate with a longer time span of the expander in the body.

Due to the smaller volume and lower projection of the expanders at the beginning of the filling period, with a more two-dimensional shape than after achievement of the final volume, it can be assumed that the risk of displacement is higher in the early phase of expansion than after complete filling.

Radiation is considered as a risk factor for complications in expander-based breast reconstruction. In a systematic review^20^ of 1501 implants, Oliver et al. described an overall explantation rate of 12% and an infection rate of 21.03% in cases of radiation before implant insertion. Kronovitz et al.^21^ found in their long-term review an overall complication rate of more than 40%; with an extrusion rate of 15 percent. We were unable to find any studies investigating the rotation behavior of expanders with different surfaces after radiation.

In our study, 11 of 74 breasts (14.9%) in the Siltex group underwent radiation after mastectomy, with rotation >45° observed in 3 cases (27%). In the TXT group, 56 breasts (52,3%) required adjuvant radiation therapy after mastectomy; 5 (8,9%) of these showed expander rotation. There was a significantly higher proportion of expanders in combination with radiation therapy in the TXT group (p < 0.001); however, this did not lead to a higher number of implant rotations.

The amount of implant defects was not significantly different (p > 0.3), with 2 out of 74 expanders in the Siltex group (2.7%) and 7 out of 107 expanders in the TXT group (6.5%).

Chemotherapy was required in 45 of the 74 expanders in the Siltex group; 61 times, chemotherapeutical procedure was necessary in the TXT group. Here too, no significant difference was found (p > 0.05), with 60,8% (Siltex) and 57,0% (TXT) respectively. It is noteworthy that 16 of 45 expanders in the Siltex group showed rotation after chemotherapy (35.6%), while in the TXT group only 7 of 56 implants with adjuvant chemotherapy (12.5%) were rotated during replacement. However, the proportion of rotated expanders in patients after chemotherapy corresponds approximately to the total proportion of implant rotation in both groups.

The extent to which patients' smoking habits influence complications associated with breast implants is a subject of controversy in the literature. The majority of studies suggest an increased risk of postoperative complications in smokers, although most studies did not investigate a correlation between the number of cigarettes smoked per day or the total duration of cigarette consumption. In the study by Kern et al^18^, a distinction was made between patients who smoked less than 5 cigarettes per day, 5-10 cigarettes per day, and > 10 cigarettes per day, with only the last group having an increased number of complications related to wound healing.

In general, an increase in revisions is seen in smokers; however, the presentation of implant loss is inconsistent.^22^ In a single-center study of 1,512 patients with 1,989 mastectomies, Kooiman et al. (23) found a 1.56-fold increased risk of reconstructive failure in smokers. None of the studies described specifically investigated the risk of expander rotation in connection with smoking. In our study, we found a history of smoking in 18 of 74 expanders (24.3%) in the Siltex group; of these, rotation was observed in 10 cases (53%). In the group with TXT expanders, 14 (13.1%) had a history of smoking, but only 2 of them (13%) showed rotation >45°. This difference appears quite striking; however, our retrospective study did not record the number of cigarettes smoked per day. The extent to which the increased rotation rate among smokers in the Siltex group is related to the surface structure of the expanders must be verified in further studies.

## Conclusion

In our study, temporary breast expanders with Siltex-surface had an increased risk of malposition with rotation greater than 45°; therefore, these expanders should preferably be used with fixation tabs.

## Funding

None.

## Ethical approval

Approval of the University of Regensburg's ethics committee (25-4330-104).

## Declaration of competing interest

Norbert Heine has served on advisory boards for Polytech Implants.
